# Bridging the Gap: Health Education Needs Among Rural Populations with Chronic Illness and Low Health Literacy in Unincorporated Communities in Southern California

**DOI:** 10.3390/ijerph23010021

**Published:** 2025-12-23

**Authors:** Shiloh A. Williams, Ryan C. Shriver, Candace C. Juhala

**Affiliations:** 1 SDSU IV RISE Center, San Diego State University–Imperial Valley, Brawley, CA 92227, USA; 2 Department of Psychology, San Diego State University, San Diego, CA 92182, USA; rshriverpearl@gmail.com; 3 School of Public Health, San Diego State University, San Diego, CA 92182, USA; cjuhala0065@sdsu.edu

**Keywords:** health literacy, health promotion, community engagement, social determinants of health, health education

## Abstract

**Highlights:**

**Public health relevance—How does this work relate to a public health issue?**

- This study examines health literacy and health information access among chronically ill adults living in rural, unincorporated communities, populations that face long standing structural, linguistic and socioeconomic health inequities.
- Findings highlight how inadequate health literacy abilities and systemic barriers to information access compound chronic disease burden in communities with limited healthcare infrastructure and minimal local governance.

**Public health significance—Why is this work of significance to public health?**

- The study provides one of the first quantitative assessments of health literacy in chronically ill individuals living in rural Southern California unincorporated communities, revealing pervasive low health literacy across demographic groups.
- Results of this study underscore that limited health literacy in these settings is likely shaped less by individual deficits and more by structural disadvantages, including low educational attainment, language barriers, poverty, and geographic isolation.

**Public health implications—What are the key implications or messages for practitioners, policy makers and/or researchers in public health?**

- Public health interventions must go beyond simple translation of materials and prioritize culturally tailored, linguistically accessible, and community-driven approaches that address underlying educational and structural inequities.
- Policies and programs should invest in system-level solutions, such as improved digital infrastructure, mobile health services, community health worker integration, and trust-building strategies, to enhance health literacy, chronic disease self-management, and healthcare access in rural, unincorporated communities.

**Abstract:**

Rural and unincorporated communities (UCs) experience persistent health disparities driven by limited healthcare infrastructure, geographic isolation, and socioeconomic inequities. Health literacy (HL), the ability to obtain, understand, and use health information, is a critical yet underexplored determinant of health outcomes in these settings. This study examined HL and barriers to healthcare and health information access among low-income adults living with chronic conditions in nine rural UCs in Southern California. A descriptive cross-sectional survey was administered in English or Spanish to 222 respondents during community food distribution events. The questionnaire included demographics, self-reported health status, chronic disease history, perceived access to care and health information, trust in information sources and HL assessment using the Newest Vital Sign (NVS). Over four-fifths (82.7%) of respondents demonstrated limited or possibly limited HL. Although Spanish-speaking respondents scored significantly lower than English speakers on the NVS, language was not a significant predictor of HL after adjusting for age, gender, education and Hispanic origin. Lower education and older age were associated with reduced HL. One in four respondents reported barriers to healthcare access, primarily due to distance and appointment availability. Over half of the respondents reported difficulty accessing or understanding health information, regardless of HL or demographic characteristics. Doctors were the most trusted source of health information, while trust in government and religious organizations was lowest. Findings reveal pervasive low HL and broad challenges accessing care and health information across rural UCs, highlighting the structural and educational inequities underlying these disparities. Addressing these gaps requires community-driven, bilingual, and culturally resonant strategies that build trust, enhance communication, and strengthen health system accessibility for residents of unincorporated rural regions.

## 1. Introduction

Rural and unincorporated communities (UCs) often face persistent healthcare disparities driven by limited access to care, fewer health resources, geographic isolation and socioeconomic inequities [1]. Rural communities are generally characterized by low population density, limited healthcare infrastructure, and significant distance from major medical and educational centers, all of which constrain access to preventive and specialty healthcare care services [1,2]. Residents of unincorporated communities, which are communities that do not have a recognized municipal or city government, face additional structural disadvantage [3]. This lack of local governance and political representation often results in exclusion and diminished access to essential resources and services, particularly for low-income communities of color who often reside in these types of communities [3,4]. Within these settings, health literacy (HL), defined as the ability to obtain, understand, and use health information to make informed decisions, is a critical yet overlooked factor influencing health outcomes [5,6]. Low HL is strongly associated with poorer management of chronic disease, increased hospitalizations, and reduced use of preventative services, compounding the burden of disease in already under-resourced populations [7,8].

### Background

Research has demonstrated a strong relationship between HL and health outcomes, particularly among individuals managing chronic conditions such as hypertension, chronic obstructive pulmonary disease, and diabetes [9,10,11]. Individuals with limited HL often struggle to navigate the healthcare system, adhere to treatment regimens, and engage in shared decision-making with providers [11].

Individuals living in rural and/or unincorporated communities (UCs), also known as Census Designated Places (CDPs), are disproportionately affected by language barriers, low educational attainment and limited access to healthcare and educational resources [1,12]. As a result, rural residents experience higher rates of preventable chronic disease incidence, reduced life expectancy, and greater difficulty accessing health services compared to their urban counterparts [7,13,14].

Barriers to health information access in rural UCs include not only physical and structural limitations, but also broader sociocultural factors [15,16,17]. Many UCs in Southern California specifically, are home to linguistically and culturally diverse populations, particularly Spanish-speaking Latino communities [3]. In these regions, limited access to language-concordant health information, materials and services, provided in the patient’s preferred language, creates significant barriers to understanding their own health needs and engaging with the healthcare team [3]. When health information is available only in English, individuals with limited English proficiency may struggle to interpret complex medical terminology, navigate healthcare systems, or follow treatment plans. This language mismatch contributes to misunderstandings, medication errors, and reduced trust in healthcare providers [18]. For individuals whose health beliefs and practices differ from mainstream models of care, these challenges may be further compounded [18,19,20,21,22]. Additionally, mistrust in healthcare systems, often prevalent among communities of color due to historical injustices, systemic discrimination, and negative personal experiences, further impedes effective health communication and engagement [23,24].

These intersecting barriers highlight the need for tailored health education strategies that account for structural, linguistic, and cultural contexts, particularly for individuals residing in rural UCs. Understanding the specific HL and health education needs of these individuals is critical to developing equitable interventions that lead to improved chronic disease outcomes and reduced health disparities within these populations. Given these interconnected challenges, this study sought to (1) describe health literacy levels among adults living with chronic conditions in rural unincorporated communities in Southern California, (2) examine sociodemographic and clinical factors associated with health literacy, and (3) characterize perceived barriers to obtaining and understanding health information, accessing healthcare services, and trusting different sources of health information. By elucidating these patterns, this study aims to inform more equitable, targeted health education strategies and system-level strategies for residents of rural unincorporated communities.

## 2. Materials and Methods

### 2.1. Study Design

This quantitative, descriptive, cross-sectional study was conducted across nine unincorporated communities (UCs) designated by the Federal Office of Rural Health Policy (FORHP) as “rural”. The study protocol, instruments, and consent procedures were reviewed and determined to be exempt by the university institutional review board (Protocol Number HS-2023-0058).

Data were collected using a structured, paper-based questionnaire administered orally and in person during monthly food distribution events organized by the local community food bank. The food bank served as a formal community partner and assisted with site coordination; however, food bank staff and volunteers did not participate in recruitment or data collection. Informed consent was obtained in each respondent’s preferred language (English or Spanish) using the approved consent form.

Because the study employed a convenience sampling approach within a small rural community context, the total number of potential respondents approached could not be systematically recorded. Therefore, a participant refusal rate was not calculated.

### 2.2. Recruitment Procedures

Respondents were recruited in person by trained community health nursing students at the food distribution events. While respondents waited in their vehicles to receive their monthly food boxes, the study team approached them at their vehicle window, introduced the study using an approved standardized script, and explained the study’s purpose, voluntary nature, and confidentiality protections.

Individuals who agreed to participate provided verbal and written informed consent prior to survey administration. For respondents accompanied by others in their vehicle, the study team offered the option to complete the survey privately in a designated tent after receiving their food box; however, all respondents chose to complete the survey in their vehicles.

This approach supported accessibility and convenience for respondents and fostered trust within the community setting while maintaining ethical standards of informed consent and confidentiality.

### 2.3. Respondents

Eligible respondents were adults 18 years of age or older who were actively enrolled in The Emergency Food Assistance Program (TEFAP) through the California Department of Social Services. Enrollment in TEFAP verified low-income status. Eligibility, including respondent age, was confirmed prior to obtaining informed consent for study participation and any individuals who were not 18 years of age or older or qualified for the TEFAP were excluded from participation in the study. All respondents completed the same bilingual (English-Spanish) questionnaire, which included demographic items (age, gender, ethnicity, and highest level of education completed), perceived health status, medical history and health literacy measures.

### 2.4. Data Collection Procedures

Data collection occurred onsite at the food bank’s monthly mobile food distribution events across nine unincorporated rural communities in Southern California from June to August of 2023. The paper-based questionnaire was administered orally to ensure accessibility for respondents with varying literacy levels and in the language of their choice. Respondents provided verbal answers and data collectors recorded responses directly onto paper forms.

Each survey took approximately 10–15 min to complete. Completed surveys were stored in sealed envelopes and transported daily to the university research office for secure storage. All identifying information was excluded to maintain respondent confidentiality.

This field-based, oral administration approach accommodated limited internet connectivity concerns at rural distribution sites and ensured participation among individuals with limited English proficiency or literacy challenges.

### 2.5. Measures

#### 2.5.1. Health Literacy Assessment

Health literacy (HL) was assessed using the Newest Vital Sign (NVS), a six-item screening tool available both in English and Spanish and developed and validated by Weiss et al. (2005) in the United States [25]. The NVS evaluates reading, numeracy, and comprehension skills using information presented on a simplified ice cream nutrition label [26,27,28]. Items 1–4 assess numeracy, or the ability to comprehend and manipulate numerical information, while items 5–6 assess document literacy, or the ability to read, interpret, and apply written health-related instructions [29]. The NVS is freely available for use in research and educational settings, and therefore no additional permission from the authors was required.

The NVS was selected for this study because it requires only about 3 min to administer. In addition, it has demonstrated high sensitivity in detecting marginal and low health literacy [24], making it well suited for use in community-based, time limited settings. The NVS has demonstrated good internal consistency (α = 0.71) and strong performance in identifying individuals with even marginally low health literacy [26].

Each correct response was scored as one point, yielding a total possible score of six [30]. Scores were interpreted as follows:

- 0–1 = high likelihood of limited/low literacy
- 2–3 = possibility of limited/low literacy
- 4–6 = adequate literacy [30].

#### 2.5.2. Perceived Health Status and Medical History

Perceived health status was measured using a single-item, five point Likert scale that has been widely used in population-based health surveys and has demonstrated validity as a predictor of morbidity and mortality [31,32]. Respondents were asked to rate their overall health as *excellent*, *very good*, *good*, *fair*, or *poor*. Responses were coded numerically (1 = poor to 5 = excellent) for quantitative analysis. Additionally, respondents were asked whether or not they had been professionally diagnosed with various common medical conditions (e.g., diabetes, hypertension, anxiety, etc.). This data allowed us to evaluate some of the potential negative health outcomes associated with poor HL.

#### 2.5.3. Accessing Healthcare Information

Respondents were asked a series of questions assessing their ability to access, understand, and evaluate health or medical information. Items measured (a) the level of effort required to obtain health information, (b) the perceived difficulty in understanding the information found, and (C) concerns about the quality or accuracy of the information. Responses were captured using a four-point Likert scale ranging from *strongly agree*, *agree*, *disagree*, and *strongly disagree*. Respondents were also asked to indicate their level of trust in various health information sources, including doctors, family, friends, government agencies (e.g., FDA, CDC), health organizations (e.g., ADA, AHD), and religious organizations. Responses were recorded on a four-point Likert scale ranging from *a lot*, *somewhat*, *a little*, and *not at all*.

#### 2.5.4. Healthcare Access

To assess potential barriers to healthcare access, respondents were asked whether they had experienced a situation in the past 12 months when they needed to see a doctor but were unable to do so (yes/no). Those who responded “yes” were asked to specify the primary reason for not accessing care (e.g., transportation difficulties, appointment availability, cost, language barriers, or other personal or structural factors).

### 2.6. Data Analysis

Of the total respondents (*n* = 222), 25 did not complete the NVS due to literacy barriers (i.e., illiterate), partial assessment completion or voluntary refusal. These individuals were excluded from analysis involving NVS scores but were retained in the dataset for descriptive analyses of demographic and health status variables. After excluding participants with missing NVS scores and conducting listwise deletion for models requiring complete demographic covariates, analytic sample sizes ranged from 164–187 depending on the model.

Statistical analyses were conducted using IBM SPSS Statistics, version 29 (IBM Corp., Armonk, NY, USA) with a statistical significance set at α < 0.05. Descriptive statistics and frequencies were calculated for all study variables.

Independent samples *t*-tests were used to examine group differences in HL scores by preferred language (English or Spanish) and chronic condition status (0–1 conditions vs. 2 or more). Potential differences in HL scores across education levels (less than high school, high school graduate, and college graduate) were examined using a one-way analysis of variance (ANOVA). In addition, a Pearson correlation was conducted to assess the association between HL scores and the total number of chronic conditions reported.

To account for potential confounding effects of demographic variables on HL scores, a multiple linear regression analysis was conducted with HL score as the dependent variable. Predictor variables included age, gender, education (less than high school, high school graduate, and college graduate), preferred language (English or Spanish), Hispanic origin (yes or no), and chronic condition status (0–1 conditions vs. 2 or more). Education was dummy-coded into two variables (high school graduate and college graduate), with “less than high school” serving as the reference category. This model assessed the unique contribution of each demographic variable to HL scores while adjusting for all others and allowed us to determine whether any group differences remained significant after accounting for all covariates.

To examine whether HL or other demographic variables were associated with respondents’ perceived effort required to obtain health information, we conducted a binary logistic regression analysis. The survey item, “*It took a lot of effort to get the information I needed*”, was dichotomized from a four-point Likert scale into *agree* versus *disagree* and used as the dependent variable. Predictor variables included HL score, age, gender, education (less than high school, high school graduate, and college graduate), preferred language (English or Spanish), and Hispanic origin (yes or no). Odds ratios (ORs) and 95% confidence intervals (CIs) were calculated to evaluate the associations between HL and perceived difficulty obtaining health information while adjusting for relevant demographic factors.

## 3. Results

### 3.1. Participant Demographics

A total of 222 individuals completed the survey. The majority (*n* = 132, 59.5%) were female and reported having Hispanic or Latinx origins (*n* = 144, 64.9%). The sample was mainly White (*n* = 95, 42.8%) or Mexican-American (*n* = 92, 41.4%), with fewer respondents identifying as American Indian (*n* = 10, 4.5%) or Other (*n* = 21, 9.5%). Language was split rather evenly, with 113 respondents reporting Spanish (50.9%) as their preferred language and 103 preferring English (46.4%). Participant ages ranged from 19 to 91 (mean age = 60.6, SD = 14.3), with over half (*n* = 144, 64.9%) indicating that their highest level of education was high school or greater (Table 1). The majority of the sample (*n* = 152, 68.5%) had previously been diagnosed with two or more chronic medical conditions (e.g., diabetes, hypertension, heart condition). Despite this, over half (59.4%) reported their health status falling between “good” and “excellent” (Table 2).

### 3.2. Health Literacy

The average HL score of the sample was 1.63 ± 1.7. Across all respondents, 113 (57.7%) had a ‘high likelihood of limited’ HL, 49 (25.0%) had a ‘possibility of limited’ HL, and only 34 (17.3%) were deemed to have “adequate” HL (Table 3). The average numeracy component (items 1–4) score across the sample was 0.88 ± 1.17, while the average document literacy (items 5–6) score was 0.74 ± 0.9. Among those who had previously been diagnosed with two or more chronic medical conditions (*n* = 152), 80 (61.1%) had a ‘high likelihood of limited’ HL, 31 (23.7%) had a ‘possibility of limited’ HL, and 20 (15.3%) were deemed to have adequate HL.

Because a significant number of respondents indicated they had one or more chronic diseases and chronic disease burden is often associated with lower HL, we compared HL scores between respondents with 0–1 chronic diagnoses and those with 2 or more. An independent samples t-test showed that respondents with 0 or 1 diagnoses had slightly higher HL scores (*M* = 1.86, *SD* = 1.75) than those with multiple conditions (*M* = 1.49, SD = 1.69), but this difference was not statistically significant, *t*(187) = 1.39, *p* = 0.167. Consistent with this finding, chronic disease burden was not correlated with HL (*r* = −0.02, *p* = 0.775). Together, these results indicate that limited HL was widespread across the sample regardless of the number of chronic conditions respondents reported.

An independent samples *t*-test initially revealed that English speakers (*M* = 2.14, *SD* = 1.83) had significantly higher HL scores compared to Spanish speakers (*M* = 1.00, *SD* = 1.40), *t*(187) = 4.73, *p* < 0.001. Additionally, a one-way ANOVA revealed a significant effect of education level on HL scores, F(2,187) = 13.63, *p* < 0.001, η^2^ = 0.13. Participants with less than a high school education (*M* = 0.78, *SD* = 1.31) had significantly lower HL scores than both high school graduates (*M* = 2.02, *SD* = 1.78), *p* < 0.001, and college graduates (*M* = 2.12, *SD* = 1.78), *p* < 0.001. HL scores did not differ between high school and college graduates, *p* = 0.715.

A multiple linear regression was conducted to examine whether our sample’s demographic characteristics predicted HL scores. The overall model was statistically significant, *F*(7,164) = 7.55, *p* < 0.001, and explained 24.4% of the variance in HL scores (R^2^ = 0.244, Adjusted R^2^ = 0.211). Consistent with our ANOVA results, education emerged as a significant predictor of HL scores. Compared to respondents with less than a high school education (reference group), high school graduates (*B* = 0.820, *p* = 0.011) and college graduates (*B* = 0.936, *p* = 0.007) had significantly higher HL scores. Age was also a significant negative predictor (*B* = −0.023, *p* = 0.011), indicating that older adults had lower HL scores. Hispanic origin was a significant predictor as well, with non-Hispanic respondents scoring higher than Hispanic respondents (*B* = 0.901, *p* = 0.029).

Importantly, preferred language (*B* = 0.037, *p* = 0.930) was not a significant predictor of HL score, nor was chronic condition status (*B* = −0.280, *p* = 0.279) or gender (*B* = 0.350, *p* = 0.150). This suggests that the initial language differences observed in the independent samples t-test were largely attributable to demographic differences between groups, particularly age, education, and Hispanic background.

### 3.3. Challenges Accessing Healthcare

A quarter of the sample (*n* = 59, 26.7%) reported difficulty seeking and accessing care from a doctor or medical professional when needed. Distance (*n* = 19) was the most frequently cited barrier to healthcare access, followed by appointment availability (*n* = 18), and inadequate transportation (*n* = 7) (Table 4).

### 3.4. Challenges Seeking Health-Related Information

In our sample, over half (*n* = 125, 56.3%) of the respondents reported difficulty accessing information about medical or health-related topics. Nearly half (*n* = 107, 48.0%) reported experiencing frustration when seeking health information, and a similar number of respondents (*n* = 112, 50.7%) expressed concern over the quality of information they were able to obtain.

### 3.5. Logistic Regression Predicting Perceived Effort to Obtain Health Information

To determine whether HL and demographic characteristics were associated with perceived difficulty obtaining health-related information, we conducted a binary logistic regression using agreement with the statement “*It took a lot of effort to get the information I needed*” as the outcome. Predictor variables included HL score, age, gender, education level, preferred language, and Hispanic origin. The overall model was not statistically significant (χ^2^(7) = 3.83, *p* = 0.799) and explained a small proportion of variance in perceived effort (Nagelkerke R^2^ = 0.03). HL score was not significantly associated with perceived effort (OR = 0.93, 95% CI: 0.76–1.13, *p* = 0.446). Age (OR = 0.99, 95% CI: 0.97–1.02, *p* = 0.481), gender (OR = 0.92, 95% CI: 0.49–1.75, *p* = 0.803), education level (all *ps* > 0.74), and preferred language (OR = 1.89, 95% CI: 0.68–5.22, *p* = 0.219) were also not significant predictors. Hispanic respondents had higher odds of reporting difficulty accessing health-related information (OR = 2.36, 95 percent CI: 0.82–6.78, *p* = 0.110), although this association did not reach statistical significance. 

### 3.6. Trusted Sources of Health Information

**Figure 1:**
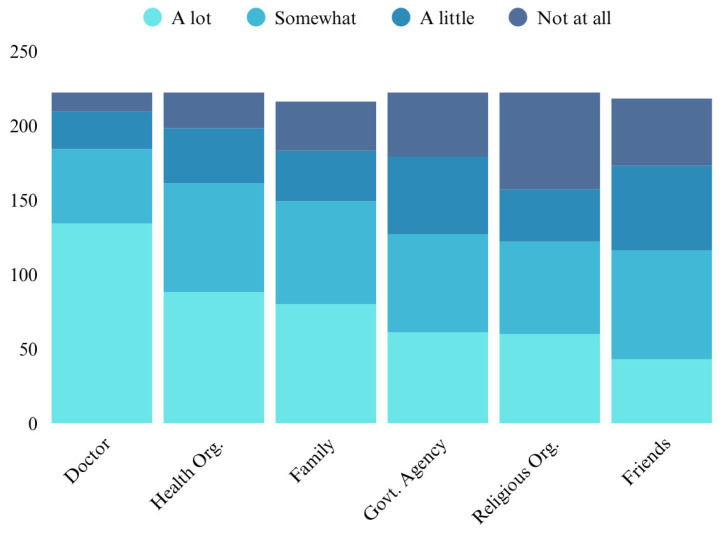
Trusted Sources of Health Information.

Respondents reported varying levels of trust in different sources of general health information (Figure 1). Doctors were the most trusted, with 60.4% of respondents indicating they trusted them “a lot” and only 5.8% reporting no trust at all. Health organizations garnered relatively high levels of trust, with 39.6% indicating “a lot” of trust. Respondents expressed greater trust in family members (37.0% “a lot”) compared to government agencies (27.5% “a lot”) and friends (19.7% “a lot”). Religious organizations were the least trusted source, with only 27.0% expressing “a lot” of trust and 29.3% reporting no trust at all.

## 4. Discussion

Consistent with prior evidence linking poor HL and adverse health outcomes [9,10], this study identified widespread limited HL among adults living with chronic conditions in rural unincorporated communities in Southern California. Over half (57.7%) of respondents demonstrated a high likelihood of limited HL, indicating that barriers to understanding and using health information are pervasive in these settings. Despite most respondents managing two or more chronic diagnoses, HL was uniformly low across all groups, suggesting that barriers to comprehension and application of health information in these communities are systemic rather than individual. These findings reinforce the need for interventions that not only expand access to healthcare services but also strengthen residents’ capacity to interpret and act on health information [9,10,11].

Although HL appeared descriptively lower among respondents with multiple chronic conditions, no statistically significant association was observed between HL and chronic disease burden. This finding suggests that, within these structurally disadvantaged communities, low HL may be a shared experience rather than an individual one. Consequently, chronic disease management challenges in this population may stem from broader social and environmental determinants, such as poverty, educational opportunity, and limited healthcare infrastructure, rather than HL alone [6,11].

Consistent with previous research [19,20], our initial analyses revealed a disparity in HL scores between English- and Spanish-speaking respondents, with Spanish speakers scoring significantly lower than English speakers on the NVS. However, when education, age, gender, chronic condition status, and Hispanic background were controlled in a logistic regression, language was no longer a significant predictor of HL. This finding suggests that the apparent language gap reflects underlying educational and socioeconomic disparities rather than linguistic ability itself. Thus, simply translating health materials into Spanish is insufficient. Effective interventions must combine linguistic accessibility with efforts to address educational inequities, literacy barriers, and socioeconomic disadvantages that limit comprehension and engagement [33].

Approximately one-quarter of respondents reported experiencing barriers to healthcare access within the past 12 months, citing travel distance, appointment availability, and cost as the most common obstacles. These barriers mirror well-documented challenges across rural America and highlight the structural isolation of unincorporated areas, which lack the governance and infrastructure resources typical of incorporated municipalities [2,17]. For residents managing chronic conditions, such access issues may exacerbate disease burden and hinder adherence to ongoing care. Expanding mobile health services, community-based screening programs and transportation support systems may help mitigate these persistent geographic and infrastructural barriers [17].

Difficulty obtaining or evaluating health-related information was also widespread., Over half of respondents reported frustration or uncertainty when seeking medical information, and many expressed concerns about the quality or credibility of available information. Importantly, HL scores and demographic characteristics were not significant predictors of perceived information-seeking difficulty, suggesting that these challenges cut across literacy and demographic lines.

This pattern likely reflects the broader structural barriers faced by individuals in rural, low-income communities [1,8]. Rather than indicating an absence of meaningful factors, these findings highlight that improving information access in such settings will require system-level solutions that address the environmental, logistical, and resource constraints shaping individual experiences [33,34]. Improving information access in rural UCs therefore requires system-level solutions, such as investment in digital infrastructure, expanding broadband connectivity, and supporting trusted community intermediaries who can bridge the gap between clinical systems and residents lived realities [34].

Trust emerged as a key factor shaping health information engagement. Respondents reported high levels of trust in doctors but considerably lower trust in government agencies and religious organizations. This pattern aligns with prior evidence documenting low institutional trust among immigrant, rural, and low-income communities, often linked to historical discrimination, perceived neglect, or fear of punitive systems, including concerns about immigration enforcement [35,36,37]. Lower levels of trust in religious organizations as sources of health information among respondents indicate that, while faith leaders may be influential within community life, they may not necessarily be viewed as credible medical authorities. Nonetheless, past research indicates that religious institutions often play multifaceted roles in public health efforts, serving both as gateways to hard-to-reach communities and as culturally grounded support systems [38].

To improve trust and engagement within these communities, healthcare systems should prioritize strengthening relationship-building and continuity of care [39]. Establishing consistent, positive interactions with the same providers can help build rapport and credibility over time embedding health literacy practices into existing infrastructure can enhance both trust and comprehension [40]. Trust can also be strengthened by disseminating reliable health information through partnerships with local institutions, such as community food banks and schools; organizations that residents already view as credible [40,41]. Additionally, training healthcare providers in cultural humility and trauma-informed care may help mitigate historical medical mistrust and promote more equitable provider-patient relationships [24].

Given the demographic composition of our sample, predominantly Hispanic and Spanish speaking, bilingual and culturally tailored strategies are especially critical. Collaborations with community health workers, *promotores de salud*, and local organizations can help ensure that information is not only linguistically accessible but also culturally meaningful and personally relevant [42]. Community-centered design approaches that involve residents in co-developing health education materials and outreach strategies can improve trust, usability and long-term sustainability of health interventions [43]. While digital and telehealth innovations hold promises for improving access, they must be deployed with sensitivity to literacy, linguistic accessibility and community trust [44].

Implementing co-designed, community-driven interventions in rural UCs present several challenges. Building and maintaining partnerships requires sustained trust, consistent communication, and equitable resource sharing between academic and community stakeholders. In communities where organizations operate with limited capacity and funding, sustaining engagement beyond the research period can be difficult. Establishing a community advisory board (CAB) composed of local residents, healthcare professionals and organizational leaders could help guide future work, ensure cultural alignment, and support continuity across projects [45,46,47] CABs can also enhance community ownership of health initiatives and foster long-term sustainability, key factors for improving health literacy and healthcare engagement in structurally disadvantaged regions [47].

### Limitations and Future Directions

Several limitations should be considered when interpreting the findings of this study. First, the use of self-reported data may introduce participant recall or social desirability bias, particularly regarding health status and information-seeking behaviors. Second, the cross-sectional design limits our ability to draw causal inferences between health literacy and health outcomes. While associations can be observed, longitudinal studies are needed to better understand these relationships. Finally, while this study provides important insights into the experiences of individuals living in rural UCs, the findings may not be fully generalizable to other populations. Contextual factors such as cultural composition, infrastructure availability, and community dynamics vary widely and may influence health communication and access in different regions.

Future research should deploy mixed-methods and longitudinal designs to better understand how health literacy interacts with trust, access, and digital inclusion in rural UCs. Qualitative inquiry could further illuminate how individuals interpret and act on health information in settings where structural inequities, language barriers, and institutional mistrust intersect.

## 5. Conclusions

This study provides one of the first quantitative examinations of health literacy levels, correlates of health literacy, and perceived barriers to accessing health information among chronically ill individuals living in rural unincorporated communities in Southern California. Findings reveal pervasive limited HL, structural and educational inequities underlying language differences, and broad difficulty accessing credible health information. Addressing these challenges requires community-driven, system-level interventions that extend beyond translation to embrace cultural relevance, trust-building, and equitable access to education and care. Efforts to improve HL in rural UCs must therefore combine the strengths of healthcare systems, communication organizations, and local residents to co-create sustainable, inclusive strategies for improving health communication.

## Figures and Tables

**Table 1:** Participant Demographics.

Demographics Variable	Respondents
Age (*n* = 222)	
18–39 years	19 (8.6%)
40–59 years	72 (32.4%)
60–79 years	116 (52.3%)
80+ years	15 (6.8%)
Gender (*n* = 221)	
Female	132 (59.7%)
Male	89 (40.3%)
Ethnicity (*n* = 219)	
Hispanic	144 (65.8%)
Non-Hispanic	75 (34.2%)
Highest Level of School (*n* = 222)	
Less than HS	78 (35.1%)
HS graduate	75 (33.8%)
College graduate	63 (28.4%)
Tech or vocational school	6 (2.7%)
Preferred Language (*n* = 221)	
English	103 (46.6%)
Spanish	113 (51.1%)
Both	5 (2.3%)

**Table 2:** Participant Self-Reported Health Status & History of Chronic Disease.

Demographics Variable	Respondents
Health Status (*n* = 222)	
Excellent	20 (9.0%)
Very Good	24 (10.8%)
Good	88 (39.6%)
Fair	70 (31.5%)
Poor	20 (9.0%)
History of Chronic Disease (*n* = 216) ^1^	
Hypertension	127 (58.8%)
Arthritis	85 (39.4%)
Diabetes	82 (38.0%)
Depression and/or Anxiety	71 (32.9%)
Lung Disease	45 (20.8%)
Thyroid	38 (17.6%)
Heart Condition	34 (15.7%)
Kidney Disease	19 (8.8%)
Other	31 (14.4%)
No prior Chronic Medical History	24 (11.1%)
Individuals Reporting 2 or More Diagnoses	152 (70.4%)

^1^ Respondents may have reported one or more conditions.

**Table 3:** Participant Health Literacy Level ^1^.

Demographics Variable	Respondents
Health Literacy Level (*n* = 196)	
Limited Likely	113 (57.7%)
Limited Possible	49 (25.0%)
Adequate	34 (17.3%)
Health Literacy Score	
Female	1.77 (SD = 1.70)
Male	1.38 (SD = 1.75)
Health Literacy Subcategory	
Numeracy	0.88 (SD = 1.17)
Document Literacy	0.74 (SD = 0.9)

^1^ As measured by the Newest Vital Sign (NVS).

**Table 4:** Difficulty Accessing Healthcare Services.

Variable	Respondents
Difficulty with access (*n* = 221)	
Yes	59 (26.7%)
No	162 (73.3%)
If yes, why? (*n* = 56)	
Distance	19 (33.9%)
Appointment availability	18 (32.1%)
Cost	7 (12.5%)
No/limited transportation	7 (12.5%)
Limited office hours	1 (1.8%)
In-office wait times	1 (1.8%)
Other reason	3 (5.4%)

## Data Availability

The raw data supporting the conclusions of this article will be made available by the authors upon request.

## Abbreviations

The following abbreviations are used in this manuscript:

NVS	Newest Vital Sign
UC	Unincorporated Communities
HL	Health literacy
CDP	Census Designated Places
